# Ultrasound findings associated with neonatal acute liver failure secondary to gestational alloimmune liver disease: A case series

**DOI:** 10.1002/jpr3.70241

**Published:** 2026-09-24

**Authors:** Naseem Ravanbakhsh, Ashley M. Upton, Chun‐Sing Huang, Nhu Thao N. Galván, John Goss, Sanjiv Harpavat, Jose A. Hernandez

**Affiliations:** ^1^ Department of Pediatrics, Division of Gastroenterology, Hepatology, and Nutrition Baylor College of Medicine and Texas Children's Hospital Houston Texas USA; ^2^ Department of Radiology Baylor College of Medicine and Texas Children's Hospital Houston Texas USA; ^3^ Department of Surgery, Division of Abdominal Transplantation Baylor College of Medicine Houston Texas USA

**Keywords:** hepatic fibrosis, non‐invasive imaging, sonographic features

## Abstract

**Objectives:**

Gestational alloimmune liver disease is the leading cause of neonatal acute liver failure, hypothesized to emerge from maternal exposure to an antigen that is expressed on fetal hepatocytes, resulting in maternal‐fetal alloimmune attack and activation of the complement cascade. Despite maximal medical therapy, some require liver transplantation for survival. Prompt recognition is vital. Ultrasound is the first imaging modality utilized in acute liver failure. Results often include nonspecific findings. There is a need for more sonographic findings to facilitate timely diagnosis. We present a case series of neonates who presented with acute liver failure secondary to gestational alloimmune liver disease at Texas Children's Hospital from 2018 to 2025.

**Methods:**

This study was approved by the Baylor College of Medicine International Review Board H40231. Medical records, imaging studies, and biopsy results from 2018 to 2025 were analyzed retrospectively. A General Electric LOGIQ E10 ultrasound machine was used.

**Results:**

All four patients with gestational alloimmune liver disease shared four findings on ultrasound: a widely patent ductus venosus, small right portal veins, reversal of flow in the right portal vein, and innumerable liver lesions.

**Conclusion:**

Our sonographic findings can be used to prompt clinical suspicion for gestational alloimmune liver disease and expedite treatment.

## Abstract (highlights)

**What is Known:**

- Gestational alloimmune liver disease is the leading cause of neonatal acute liver failure.
- Ultrasound is often the first imaging modality utilized in acute liver failure.
- A persistently patent ductus venosus has been reported on ultrasound in neonates with gestational alloimmune liver disease.
- A patent ductus venosus can also be seen in prematurity and with other congenital anomalies.

**What is New:**

- We identified a constellation of features among four neonates with acute liver failure from gestational alloimmune liver disease, including a widely patent ductus venosus, small right portal veins, reversal of flow in the right portal vein, and innumerable liver lesions.

## 1 INTRODUCTION

Neonatal acute liver failure (ALF) is a rare disease associated with high morbidity and mortality, commonly the result of viral infections, hemophagocytic lymphohistiocytosis (HLH), mitochondrial hepatopathies, and gestational alloimmune liver disease (GALD).^1^

GALD is the leading cause of neonatal ALF, hypothesized to emerge from maternal exposure to a sensitizing antigen that is expressed on fetal hepatocytes, resulting in maternal‐fetal alloimmune attack and activation of the complement cascade. Consequently, developing hepatocytes are injured, which can manifest as ALF or cirrhosis. Extrahepatic siderosis on magnetic resonance imaging (MRI) or buccal biopsy are suggestive of GALD.^2^ Treatment includes double volume exchange transfusion (DVET) and intravenous immunoglobulin G (IVIG).^2^, ^3^ Despite medical therapy, a subset of neonates requires liver transplantation (LT) for survival.

Prompt recognition is vital, as delayed treatment risks irreversible hepatic injury and death. Ultrasound is the first imaging modality utilized in ALF, though results are often nonspecific and may include abnormal hepatic echogenicity, “starry sky” appearance, and gallbladder wall edema.^4^ In HLH, findings may include ascites, increased periportal echogenicity, gallbladder wall thickening, and hepatosplenomegaly, the latter reflecting infiltration of lymphocytes and histiocytes in the reticuloendothelial system.^5^ Mitochondrial hepatopathies may present with hepatic steatosis.^6^ Prior case series have observed a persistently patent ductus venosus (DV) on imaging in GALD.^2^, ^7^ Notably, the DV typically closes within the first week of life in most healthy infants.^8^ There is a need for more sonographic findings to facilitate timely diagnosis of GALD.

Herein, we present a case series of four neonates who presented with ALF at Texas Children's Hospital (TCH) from 2018 to 2025 secondary to GALD, all of whom shared characteristic findings on ultrasound. We also included the ultrasound findings of neonates who presented with acute liver failure from non‐GALD etiologies for comparison.

## 2 METHODS

### 2.1 Ethics statement

This study was approved by the Baylor College of Medicine International Review Board H40231. Medical records, imaging studies, and biopsy results from 2018 to 2025 were analyzed retrospectively. The requirement for informed consent was waived given the retrospective nature of the study.

### 2.2 Ultrasound

A General Electric LOGIQ E10 ultrasound machine was used. A linear, high‐frequency probe, defined as at least 15 megahertz or higher, was used to identify liver lesions. Ultrasounds were interpreted by fellowship‐trained pediatric radiologists.

To compare the ultrasound findings in our four GALD cases, we selected a comparison cohort of neonates based on recall by study personnel who also presented with acute liver failure from 2019 to present who had undergone ultrasound. We specifically included patients with diagnoses other than GALD.

## 3 RESULTS

### 3.1 Case presentations

#### 3.1.1 Patient 1

The patient was born at 36 weeks to a G2P1 mother. Pregnancy was complicated by gestational diabetes and threatened miscarriage. The neonate was small for gestational age and microcephalic. Following delivery, the patient developed hypoglycemia, hyperbilirubinemia, and elevated transaminases. Buccal biopsy demonstrated iron deposition in the acinar cells. Ferritin was 2770 ng/mL. Infectious studies and whole genome sequencing (WGS) were negative. Ultrasound demonstrated a patent DV, small right portal veins (PVs), reversal of flow in the right PV and left PVs distal to the DV, and innumerable liver lesions (Table 1). The patient was treated with IVIG and DVET. The patient was denied for transplant due to small size.

**Table 1:** Sonographic findings, individual and shared, among neonates with GALD.

Patient	US (DOL)	Vascular findings (specific)	Vascular findings (shared)	Parenchymal findings (shared)
1	22	Reversal of flow in the left PVs distal to the ductus venosus	Widely patent DV Small right PVs Reversal of flow in the right PV Normal main PV with hepatopetal flow All portal flow shunted into the systemic circulation via the DV, bypassing the liver	Innumerable round liver nodules/lesions
2	27	Patent umbilical vein		
3	17	Reversal of flow in the left PVs distal to the ductus venosus		
4	17	Reversal of flow in the left PVs distal to the ductus venosus		

Abbreviations: DOL, day of life; DV, ductus venosus; GALD, gestational alloimmune liver disease; PV, portal vein(s).

#### 3.1.2 Patient 2

The patient was born at 34 weeks to a G8P4 mother. Pregnancy was complicated by decreased fetal movement and intrauterine growth restriction (IUGR). The patient developed bleeding from the umbilical cord, labs significant for elevated liver enzymes and coagulopathy. Due to the concern for GALD, the patient was treated with IVIG and DVET without clinical improvement. Workup was significant for a negative buccal biopsy, ferritin of 1230 ng/mL, negative infectious studies, and an unremarkable WGS, which demonstrated pathogenic variants in the *ABCB4* and *SKIC2* genes. Ultrasound demonstrated a patent DV, small right PVs, reversal of flow in the right PV, patent umbilical vein, and innumerable liver lesions (Table 1). The patient was transferred to another institution and underwent LT at 16 weeks of life. Diagnostic uncertainty remains, given lack of extrahepatic siderosis in the diagnostic workup.

#### 3.1.3 Patient 3

The patient was born at 36 weeks to a G1P0 mother. Pregnancy was complicated by IUGR, oligohydramnios, and decreased fetal movement. The patient developed hyperbilirubinemia and coagulopathy, and was treated with IVIG and antibiotics. Buccal biopsy on the second day of life demonstrated iron deposition in salivary gland acinar epithelial cells, suggestive of GALD. The patient was treated with DVET. Ferritin was 5520 ng/mL. Infectious workup was negative and WGS unremarkable with several variants in *HFE*, *PCCA*, and *TNFRSF1A* genes. Ultrasound demonstrated a patent DV, small right PVs, reversal of flow in the right PV and left PVs distal to the DV, and liver lesions (Table 1). The patient underwent LT at 8 weeks of life.

#### 3.1.4 Patient 4

The neonate was born at 39 weeks to a G2P1 mother with a history of a molar pregnancy. Birth was complicated by the presence of meconium. The patient had generalized edema, coagulopathy, hypoalbuminemia, as well as ascites and heterogeneous hepatic parenchymal echogenicity on ultrasound. Echocardiogram within the first days of life demonstrated a bidirectional patent ductus arteriosus, flattened septum, patent foramen ovale with left to right shunting, and elevated right ventricular pressure, concerning for pulmonary hypertension. MRI showed extrahepatic deposition in the thyroid, suggestive of GALD. The patient was given IVIG followed by DVET without clinical improvement. Ferritin was 4180 ng/mL. Infectious workup and whole exome sequencing were negative. Ultrasound demonstrated a patent DV, small right PVs, reversal of flow in the right PV and left PVs distal to the DV, and liver lesions (Table 1). The patient underwent cardiac catheterization at 3 weeks of life, which showed normal pulmonary vascular resistance. The patient underwent LT at 4 weeks of life.

### 3.2 Comparison cohort

**Figure 1:**
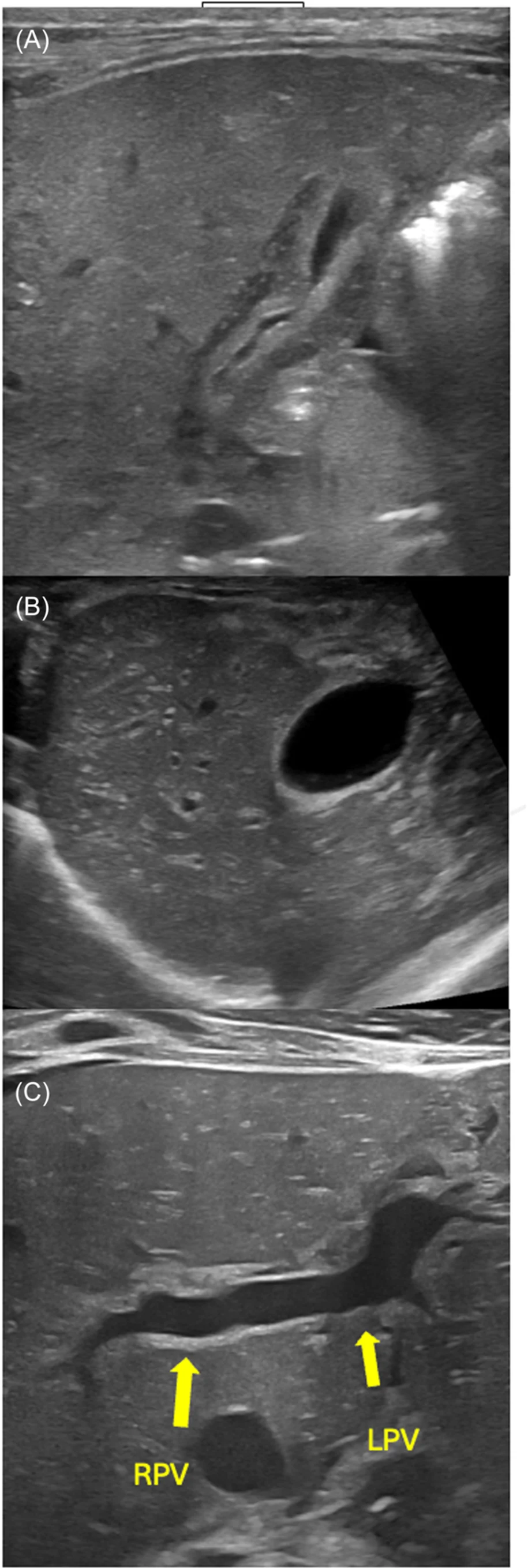
(A) Sagittal image of an incompletely distended gallbladder with pericholecystic fluid (B) Increased periportal echogenicity or “starry sky” appearance of the liver with diffusely hypoechoic liver parenchyma (C) Normal sized intrahepatic portal veins (RPV and LPV labeled with yellow arrows). LPV, left portal vein; RPV, right portal vein.

We present five neonates with ALF from etiologies other than GALD: Patient A was found to have disseminated herpes simplex virus‐2 infection; ultrasound at 3 weeks of life demonstrated heterogeneity of liver parenchyma with a nodular contour (no hepatosplenomegaly; non‐patent ductus venosus, and the patient had normal‐sized PVs) and gallbladder wall edema (Figure 1A). Patient B was diagnosed with familial HLH due to *PRF1* compound heterozygous variants; ultrasound at 6 days of life showed hepatomegaly and increased periportal edema (otherwise normal appearing spleen, non‐patent ductus venosus, normal‐sized PVs). Patient C had suspected HLH on autopsy; ultrasound at 2 weeks of life demonstrated gallbladder edema, increased peri‐portal echogenicity or “starry‐sky appearance” of the liver (Figure 1B) and a patent ductus venosus, and normal‐sized PVs (Figure 1C). Patient D was diagnosed with OTC deficiency; ultrasound at 1 week of life demonstrated a heterogenous echotexture of the liver, patent ductus venosus (otherwise normal appearing spleen and PVs). Patient E was diagnosed with infantile hemangiomas; ultrasound at 2 weeks of life demonstrated large hemangiomas, hepatomegaly, a patent ductus, and a normal appearing spleen.

## 4 DISCUSSION

GALD has been described on cross‐sectional imaging, histopathology, and supported by certain biochemical markers. To date, ultrasound findings beyond a patent DV have not been described. Notably, there remains some diagnostic uncertainty for GALD in patient two, given the negative buccal biopsy, though it was the leading differential given ALF with unremarkable genetics and infectious studies. GALD is a challenging diagnosis to make in a timely manner. The evaluation poses barriers for neonates who have blood volume limits due to size. MRI and biopsy require anesthesia which have risks and require transportation of critically ill patients who are reliant on extracorporeal liver systems and vasoactive medications. The feasibility of ultrasound, which we observed has parenchymal and vascular findings characteristic of GALD, can help facilitate early clinical suspicion. Our constellation of sonographic findings in patients with GALD were not present in neonates who presented with ALF from other etiologies including viral infection, HLH, urea cycle disease, and infantile hemangiomas.

**Figure 2:**
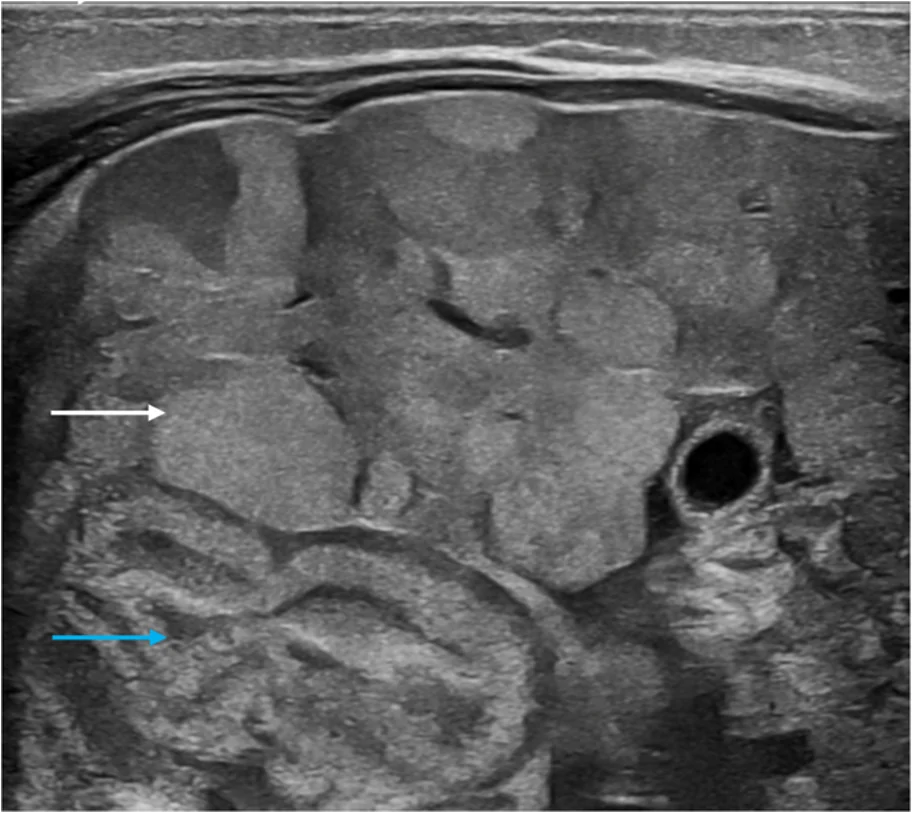
Transverse ultrasound image of the right liver (white arrow) and kidney (blue arrow) using a high‐frequency (15 MHz) linear transducer demonstrating innumerable round liver nodules of varying echogenicities, forming an irregular liver capsule.

Parenchymal findings using ultrasound include hepatic lesions. Histologically, GALD has been described as the presence of parenchymal neotubules, lack of mature hepatocytes, and hepatic fibrosis.^9^ While findings are smaller than what ultrasound is able to decipher, we hypothesize that the assortment of microscopic features manifest sonographically as disarray distortion of the liver parenchyma, which appear as countless hepatic lesions and nodules (Figure 2).

**Figure 3:**
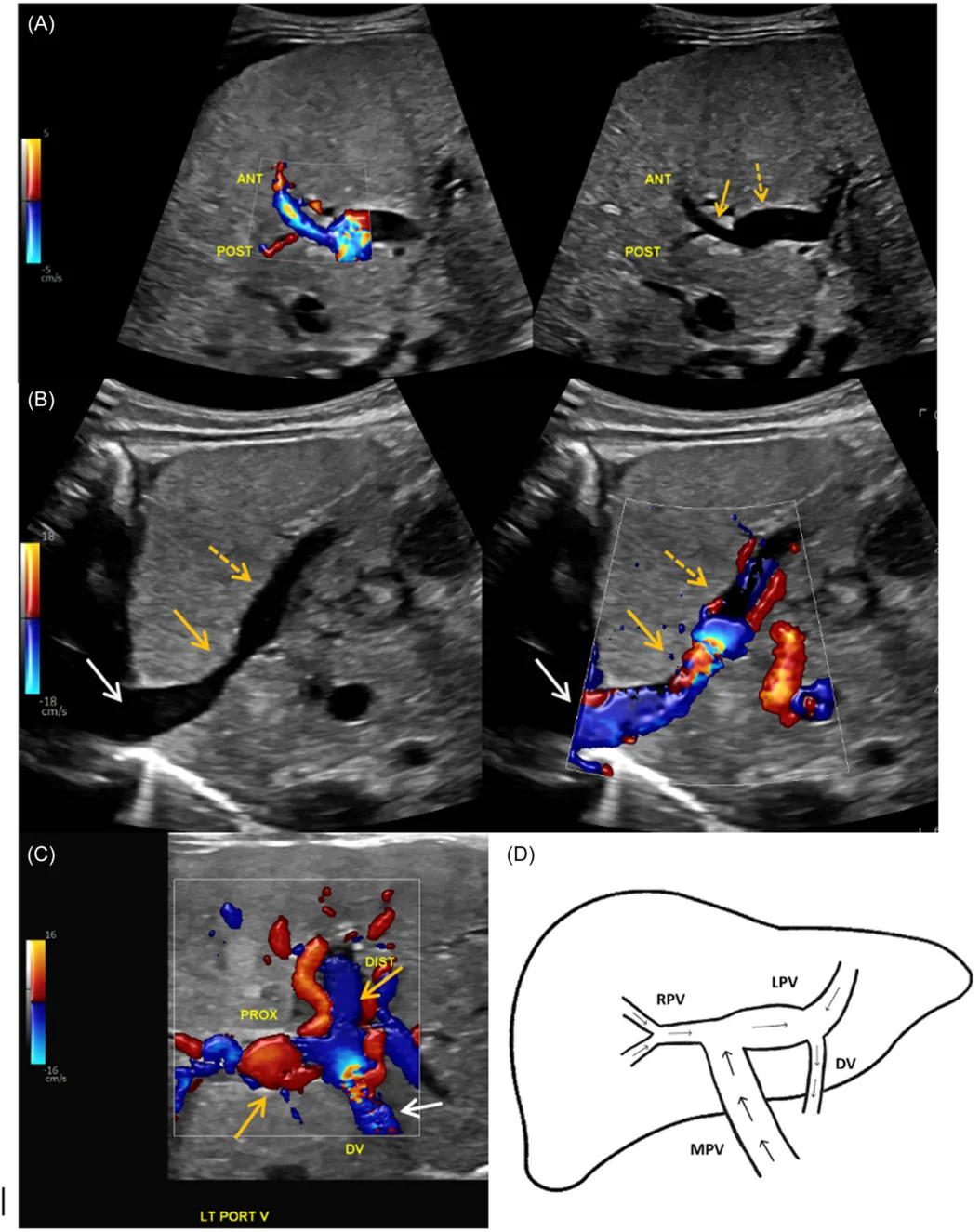
(A) Dual screen color doppler and grayscale transverse image demonstrates the size discrepancy of the intrahepatic PVs. The right PV (solid arrow) has hepatofugal blood flow and is significantly smaller in size compared to the central LPV (dashed arrow) before blood flow is shunted into the patent DV. (B) Sagittal grayscale and color Doppler image of the widely patent DV (solid orange arrow) with hepatofugal blood flow from the LPV (dashed line) diverting to the IVC (solid white arrow) (C) Transverse view of the LPV (orange arrows) with hepatopetal flow (red) proximal to the DV and hepatofugal flow (blue) distal to the DV (white arrow) (D) Schematic diagram of hepatic vasculature correlating with ultrasound images in the transverse plane. DV, ductus venosus; IVC, inferior vena cava; LPV, left portal vein; MPV, main portal vein; PV, portal vein; RPV, right portal vein.

Vasculature findings include abnormalities in the portal venous system (Figure 3A,C) and a patent DV (Figure 3B). A patent DV has been described as a pathophysiologic attempt to alleviate elevated portal pressure in a case series of three infants with GALD.^7^ We speculate that because no other cause of ALF begins with targeted destruction of hepatocytes in utero with subsequent development of portal hypertension, a persistently patent DV may be characteristic of GALD. Abnormal features of the portal venous system may be rooted in the same pathophysiology that causes the DV to maintain patency. We propose two possible mechanisms: while hepatopetal blood flow is normally maintained through the main PVs, the intrahepatic portal venous blood flow meets resistance due to fibrosis and re‐routes through the DV, a path of less resistance; diminished blood flow through the small right intrahepatic PVs prevents their development, explaining their small appearance on ultrasound (Figure 3A). Alternatively, the liver and PVs do not develop properly in GALD, and blood flow is diverted elsewhere, through the DV.

As mentioned, the study by Tsai et al. previously identified a patent DV as a feature of GALD but did not report the constellation of findings we observed in our case series. We postulate that because Tsai et al.'s study is over 15 years old, we observed additional findings of the portal venous system and hepatic parenchyma in patients with GALD as a result of updated software and technology that produced more sensitive imaging of the liver; further, while their study did not comment on the type of ultrasound that was used, limiting our ability to directly compare our findings, our study utilized General Electric LOGIQ E10, which was only made available in 2018, years after their case series. The high‐frequency probe identified the liver lesions, and such modality was not mentioned in the study by Tsai et al.

A limitation of our study is that it did not have a complete comparative sample, and thus, it is possible that these features are not unique to GALD. Future prospective studies with a larger cohort of neonates are needed to verify our findings.

## 5 CONCLUSION

Our constellation of sonographic findings can be used to prompt clinical suspicion for GALD and expedite treatment. All neonates demonstrated four shared features on ultrasound: a widely patent DV, small right PVs, reversal of flow in the right PV, and innumerable liver lesions.

## CONFLICT OF INTEREST STATEMENT

The authors declare no conflicts of interest.
